# Vector-Borne Disease Intelligence: Strategies to Deal with Disease Burden and Threats

**DOI:** 10.3389/fpubh.2014.00280

**Published:** 2014-12-22

**Authors:** Marieta Braks, Jolyon M. Medlock, Zdenek Hubalek, Marika Hjertqvist, Yvon Perrin, Renaud Lancelot, Els Duchyene, Guy Hendrickx, Arjan Stroo, Paul Heyman, Hein Sprong

**Affiliations:** ^1^Centre for Zoonoses and Environmental Microbiology, Netherlands National Institute for Public Health and the Environment (RIVM), Bilthoven, Netherlands; ^2^Medical Entomology Group, MRA, Emergency Response Department, Public Health England, Salisbury, UK; ^3^Medical Zoology Laboratory, Institute of Vertebrate Biology, Academy of Sciences, v.v.i., Brno, Czech Republic; ^4^Faculty of Science, Department of Experimental Biology, Masaryk University, Brno, Czech Republic; ^5^Public Health Agency of Sweden (Folkhälsomyndigheten), Solna, Sweden; ^6^Centre National d’Expertise sur les Vecteurs, Centre IRD de Montpellier, Montpellier, France; ^7^CIRAD, UMR CMAEE, Montpellier, France; ^8^INRA, UMR CMAEE 1309, Montpellier, France; ^9^Avia-GIS, Zoersel, Belgium; ^10^Centre for Monitoring of Vectors, Netherlands Food and Consumer Product Safety Authority (NWVA), Wageningen, Netherlands; ^11^Research Laboratory for Vector-Borne Diseases, Queen Astrid Military Hospital, Brussels, Belgium

**Keywords:** vector-borne diseases, surveillance, one health, disease burden, threat, emerging diseases

## Abstract

Owing to the complex nature of vector-borne diseases (VBDs), whereby monitoring of human case patients does not suffice, public health authorities experience challenges in surveillance and control of VBDs. Knowledge on the presence and distribution of vectors and the pathogens that they transmit is vital to the risk assessment process to permit effective early warning, surveillance, and control of VBDs. Upon accepting this reality, public health authorities face an ever-increasing range of possible surveillance targets and an associated prioritization process. Here, we propose a comprehensive approach that integrates three surveillance strategies: population-based surveillance, disease-based surveillance, and context-based surveillance for EU member states to tailor the best surveillance strategy for control of VBDs in their geographic region. By classifying the surveillance structure into five different contexts, we hope to provide guidance in optimizing surveillance efforts. Contextual surveillance strategies for VBDs entail combining organization and data collection approaches that result in disease intelligence rather than a preset static structure.

## Introduction

Globalization and human population growth continue to put increasing pressures on human health and well-being. Emerging diseases have become a growing threat leading to the development of epidemic intelligence systems to aid public health authorities. The main aim of this epidemic intelligence is to encompass all activities that permit the early identification of potential health hazards, their verification, risk assessment, and investigation in order to inform and improve public health control measures in a timely manner (1). Epidemic intelligence integrates both an indicator-based and an event-based component; the former referring to structured collection of data through routine surveillance systems and the latter referring to data gathered from sources of intelligence of any nature (2). Finally, good epidemiologic judgment, the reasoning process that indicates when there is sufficient data on which to make public health decisions (1), is essential. This has been a challenging component in the past but even more so today with mandated transparency of public decisions, and a continuously changing interconnected world. Further, it has become apparent that an interdisciplinary approach to the prevention and control of zoonoses is invaluable. Cross-sector working ensures better preparedness and contingency planning, more efficient and effective surveillance systems, cost-sharing between sectors according to their benefits of control, increased health equity, and improved sharing of logistics and costs for service provision (3). In recent years, efforts to improve the collaboration between the public and veterinary health, has paid off (4). Since the transmission cycles of several zoonotic pathogens occur in nature, involvement of stakeholders of forest and nature management is a logical next step. This means combining organization and data collection approaches that result in disease intelligence (2) rather than a preset static structure.

**Figure 1:**
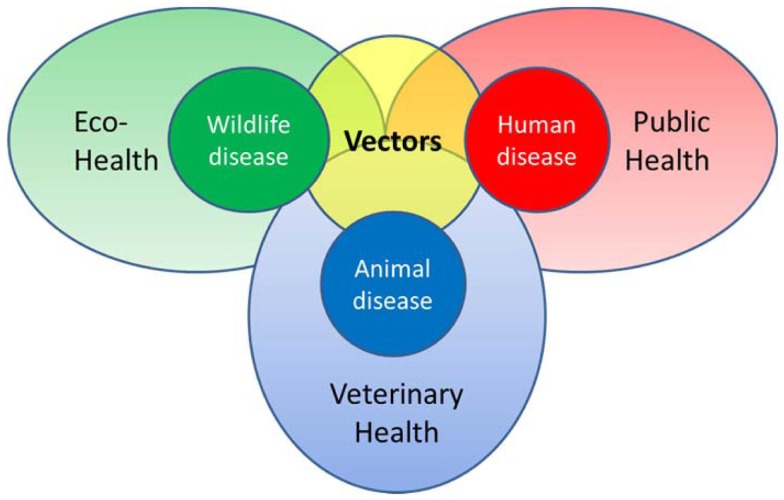
**Schematic representation of the various health disciplines involved in vector-borne diseases**.

In recent decades, vector-borne diseases (VBDs) have emerged as a significant threat to human health in temperate areas (5). In Europe, the incidence and geographical distribution of endemic VBDs, specifically Lyme borreliosis, are increasing in several areas (6). Also, West Nile fever is emerging in Europe (7). Other VBDs have appeared outside the regions where they originally circulated such as dengue in Madeira (8). And last but not least, novel VBDs have emerged or have recently been recognized, such as infections with the tick-borne *Borrelia miyamotoi* (9). Considering the already occurring disease burden and various emerging threats for the future, cost-effective VBD surveillance is challenging. Investment in networks keeping a close watch on the matters within their expertise, public health, veterinary, and ecohealth (Figure 1) is needed. Here, ecohealth is defined as the combined health effects of (changes to) the ecologic network in which we function. This also includes the implications of nature management decisions and land use on factors influencing VBD epidemiology. Each and every pathogen has its own disease ecology; therefore, the combined effect of changes affecting factors like, for instance, reservoir species distribution, vector densities, and human exposure, is inheritably complex.

We aim to develop a strategy for the surveillance of vector-borne pathogens, on a national as well as a Pan-European context. When developing and structuring VBD surveillance strategies, consideration of potential prospectives for action is important. Here, we present the building blocks of such contextual surveillance strategy. We expand the surveillance pyramids conventionally used as the basis of public health surveillance to pyramid assemblies, encompassing data from livestock (and pets), wildlife, and vector population. Next, we customize the pyramid assemblies to specific diseases, here focusing on those transmitted by mosquitoes and ticks. The contextual surveillance strategy of VBDs is further illustrated using examples from the Netherlands. Finally, we link the national with Pan-European VBD surveillance strategies.

## Building Blocks

### Population-based surveillance

**Figure 2:**
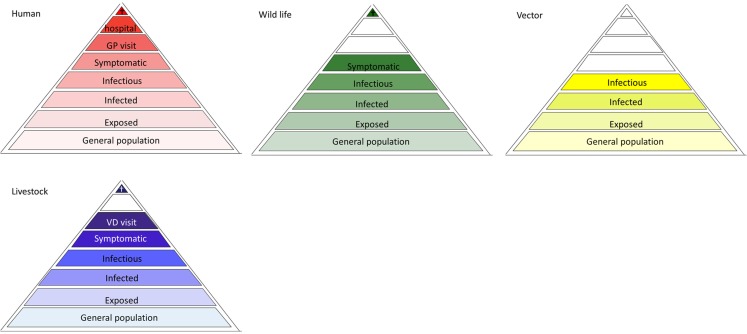
**Surveillance pyramid assembly of a hypothetical vector-borne zoonosis**. Not all levels exist in surveillance pyramid of all populations. The color scheme used refers to Figure 1.

To adequately monitor the disease burden of VBDs related to their occurrence, information is needed from different subgroups of the human population, represented by a disease burden pyramid. However, the forecasting, early detection, prevention and control of VBDs requires knowledge of other parameters. Acquiring data on the presence and density of (infected) vectors complements the disease burden pyramid for VBDs. In the case of a zoonotic VBD, an additional level is required to encompass the density of (infected) reservoir hosts (10). Actually, for each of these layers (10), a surveillance pyramid of their own can be drawn, which can be used when developing a contextual surveillance strategy. Separate surveillance pyramids can also be drawn for each of the additional population groups (e.g., sentinels or affected animal populations), highlighting all possible targets and levels of surveillance, forming a surveillance pyramid assembly (Figure 2). The distinction between wild and domestic animals is necessary to match the different health disciplines involved in the data collection (see also Figure 1). For all pyramids, the level of “infectious population” is added to better assess transmission risks. While all surveillance pyramids have the same organization, not all levels occur in every pyramid. For example, the top four surveillance levels of the pyramid do not exist for vectors. An additional point is that some pyramids might be absent in the surveillance pyramid assembly (see below).

### Disease-based surveillance

**Figure 3:**
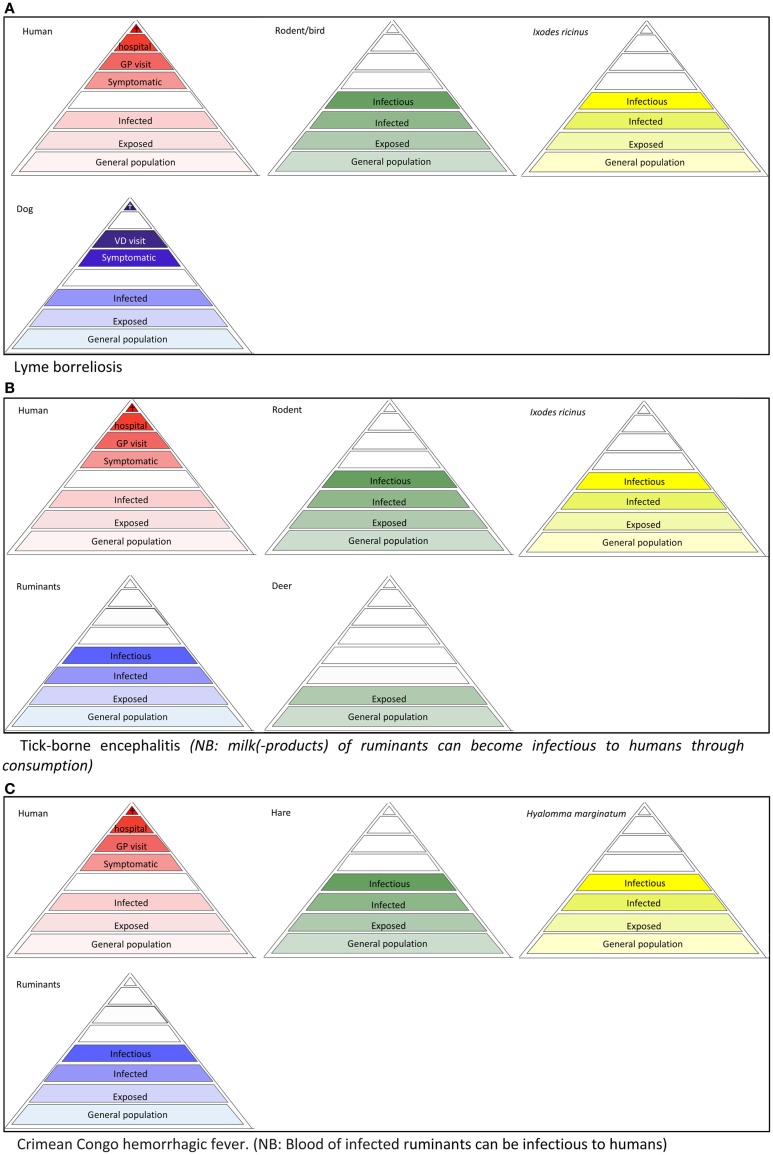
**Disease depended surveillance pyramids of tick-borne diseases, (A) Lyme borreliosis (B) tick-borne encephalitis, (C) Crimean Congo hemorrhagic fever**.

**Figure 4:**
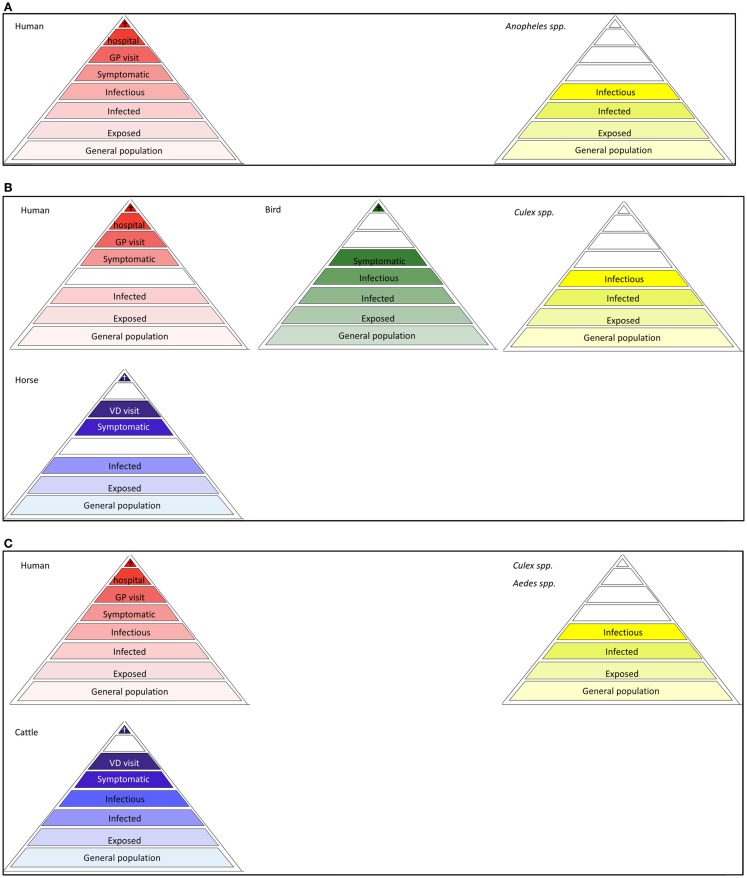
**Disease dependent surveillance pyramids of mosquito-borne diseases (A) Malaria, (B) West Nile fever, (C) Rift Valley fever**. The pyramid assemblies for dengue and chikungunya resemble that of malaria, provided that *Anopheles* spp is replaced by *Ae. albopictus* and *Ae. aegypti* as vector.

To accurately describe all possible targets of surveillance for a specific VBD, tailoring the surveillance pyramid assembly is important. Some vector-borne pathogens, such as malaria, dengue, and chikungunya, can be maintained in a human-vector-human cycle, with no involvement of animals in the transmission cycle. Most vector-borne pathogens, however, circulate among vectors and (wild) reservoir animals. Here, humans frequently act as dead-end hosts, from which pathogens are not transmitted to other susceptible hosts, in nature; non-natural routes of transmission (e.g., through blood transfusion) or “rare” routes (e.g., mother-to-child transmission) are not considered here. In these cases, the infectious level is omitted from the human surveillance pyramid (Figures 3A–C and 4B). The same holds for animal species that are dead-end hosts (Figures 3A and 4B). In some cases, the addition of a pyramid for animals, that are neither a reservoir nor a host, but may serve as a sentinel for exposure, can be useful. Spill-over of tick-borne encephalitis virus, for example, has been revealed in dogs and deer in Belgium (11, 12). Similarly, chickens may serve as sensitive sentinels for West Nile virus (WNV) without contributing to the virus transmission cycle. Vector surveillance usually entails data on the population sizes and the infection rates.

### Context-based surveillance

The concept of surveillance pyramid assemblies offers a systematic approach for identifying all possible targets and levels of surveillance of any VBD. The next step is to develop a cost-effective strategy for surveillance for VBD, which largely depends on the purpose of the surveillance. Selecting the particular layers of each population to include as sensible targets for surveillance depend on the contexts of VBDs. A surveillance strategy should be based on the identification of the involved levels for a certain disease and the technical/laboratory methods (defining the feasibility) that can support the surveillance of that level (10).

The presence of a vector is a prerequisite for the transmission of a VBD. While the absence of a vector prevents transmission, the presence of a vector does not imply that actual transmission of a pathogen is occurring or will occur in the future. Based on this premise, the status of a VBD can be described in five different contexts based on the presence or absence of the three facets of VBDs important for public health: human cases, pathogens, and vectors (Table 1) (10). In short, VBDs with disease burden in a country fall under context 1 and the remainder fall under one of the other four contexts (Contexts 2–5).

**Table 1:** **Current status of vector-borne diseases in the Netherlands**.

Context	Disease burden^1^	Pathogen^2^	Vector	Tick-borne pathogens	Mosquito-borne pathogen
1a	√ (every year)	√	√	*Borrelia burgdorferi spp*.	No examples
1b	√ (not every year)	√	√	*Borrelia miyamotoi*	No examples
2	–	√	√	*Rickettsia helvetica, Neoehrlichia mikurensis, Anaplasma phagocytophilum, Ehrlichia & Babesia, Bartonella*	*Plasmodium* spp
3	–	–	√	Tick-borne encephalitis virus	*Dirofilaria* spp., Mosquito-borne viruses (Batai, Inkoo, Rift Valley, Sindbis Snowshoe hare, Tahyna, Usutu, West Nile)
4	–	√	–	*Coxiella burnetti, Francisella tularensis*	Chikungunya virus; Dengue virus
5	–	–	–	Crimean Congo Hemorrhagic Fever virus	Japanese encephalitis virus^3^

*^1^Locally acquired human case*.

*^2^Imported human case, infected animal reservoir, or vector*.

*^3^Potentially European mosquitoes are competent to transmit JEV (13), but this has not been validated*.

To identify, assess the risk, communicate, and ultimately control VBDs, monitoring and surveillance tools, appropriate to the context, are needed (10). Generally, the current impact of a health problem is assessed through burden of disease calculations, while the impact of future outbreaks in an area is determined through quantitative risk assessments. Although applicable for endemic VBDs (context 1, Table 1), such quantitative assessments would not be sensible for the remaining VBD contexts without actual disease burden. Nevertheless, information on these so-called threats to human health is desirable (10). Consequently, surveillance efforts for VBDs, that are endemic and cause disease burden, need to have a different focus and structure than those for VBDs that are a threat. As a consequence, any surveillance strategy for a VBD needs to be developed considering its context.

## National Context

When comparing tick-borne with mosquito-borne diseases in Netherlands (Table 1), for example, the most striking difference arises in context 1, i.e., endemic diseases. While the disease burden of mosquito-borne diseases is currently zero, the disease burden of tick-borne diseases, such as Lyme disease, is significant and growing (14). In the Netherlands, an increase in reported cases of tick bites and erythema migrans, the first clinical symptom of Lyme disease, has been reported over the last 15 years (14, 15).

For mosquito-borne diseases in the Netherlands, risk estimates, preparedness, and early warning are the more important components of risk analyses (10). Risk estimates are very useful in providing insight into the complexity of VBD emergence, provided that their assumptions, uncertainties, and ambiguities are taken into account (16, 17). Between 30 and 40 species of mosquito are indigenous to north-west Europe, many of which act as potential vectors of pathogens. However, since the eradication of malaria in the 1960s, no mosquito-borne diseases have been reported. Still, several endemic mosquito species are proven vectors of zoonoses or malaria in the laboratory or in the field elsewhere in Europe. With this in mind, various mosquito-borne diseases are categorized as context 3. Whether actual transmission of mosquito-borne pathogens occurs after introduction depends upon the vector capacity. The consequences of pathogen introduction in a region with a competent mosquito vector population can be very quick and significant, as illustrated with WNV introduction in the United States in 1999 (18) or chikungunya virus introduction in the Caribbean in 2013 (19). Development of contingency plans (including outbreak exercises) for potential emergence and databases with background information are tools to improve preparedness. For early warning purposes, focus is placed on improving and updating detection tools for the laboratory (20) and field (21) to enable rapid pathogen detection in a vector, reservoir, or host, when introduced. To this end, knowledge of the contexts of VBDs in the rest of Europe and more globally through international collaboration and networks is essential to enhance preparedness. This also applies to non-endemic tick-borne pathogens (22).

Here, we further describe surveillance strategies when placed in context, in reverse order (Contexts 5–1). Overviews of surveillance strategies for a mosquito- and tick-borne example (when available) in the Netherlands are shown in a box.

Context 5 deals with VBDs that currently do not pose any risk to the country owing to the current absence of both the pathogen and vector. The main concern centers therefore on the future establishment of the vector upon its introduction. Surveillance of pathogens and/or human cases is not a priority at this stage. If vector establishment on the basis of climatic or environmental constraints is impossible or highly improbable, no surveillance activities are recommended. However, this assessment needs to be iterative accounting for changes in the geographic distribution of the vectors in Europe.

**Table:** 

**Figure 5:**
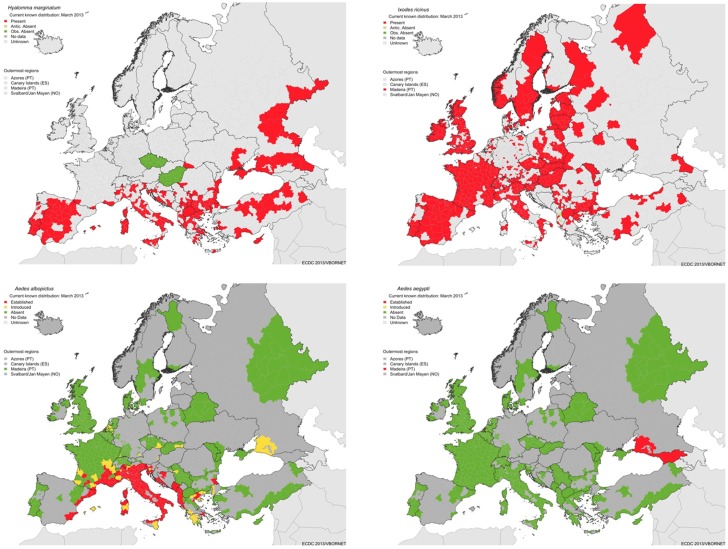
**Geographic distribution of major vectors in Europe: ticks: *Hyalomma marginatum*, vector of Crimean Congo hemorrhagic fever (top left), *Ixodes ricinus*, vector of Lyme borreliosis and tick-borne encephalitis (top right), and *Ae. albopictus* (bottom left) and *Ae. aegypti* (bottom right), vectors of dengue (source: ECDC)**.

**Japanese encephalitis risk to the Netherlands (Context 5)** Japanese encephalitis is endemic to South East Asia. The principal vectors are *Culex* mosquitoes, and to a lesser degree, *Aedes*. The main vector in Asia, *Cx. tritaeniorhynchus*, is not present in Europe. Recently, an ambiguous report on the detection of fractions of this virus in *Cx. pipiens* in Italy appeared (13). Until there is conclusive evidence of Japanese encephalitis virus in Europe, this virus and its main vector are considered absent in Europe (23). Therefore, no surveillance activities are recommended.
**Crimean Congo Hemorrhagic fever risk to the Netherlands (Context 5)** The main vector of Crimean Congo hemorrhagic fever virus is the hard tick *Hyalomma marginatum* and its current known European distribution is shown in Figure 5. Transmission only occurs in regions within the vector’s geographic distribution. These ticks have been occasionally imported to Northwestern Europe on migratory birds (24, 25). Modeling suggests that the current climate of the Netherlands is unsuitable to sustain a population of these ticks, with little prospect of their establishment in the foreseeable future (26, 27), making any surveillance activity in the surveillance pyramid assembly obsolete. Nevertheless, source-finding activities in response to incidental findings of specimen to confirm absence of a local population should be considered.

Context 4 deals with VBDs where although the vector is absent, the pathogen is regularly introduced. In comparison to context 5, owing to the introduction of the pathogen, assessments whether or not the vector can establish, upon introduction, has priority. If local climatic and environmental conditions permit establishment, surveillance focusing on detecting the introduction of the vector at potential ports of entry/risk locations is required. Only the vector pyramid within the surveillance pyramid assembly needs to be considered, preferentially also in an international context.

**Table:** 

**Dengue virus risk in the Netherlands (Context 4) – excluding current risks in the Dutch Overseas Territories** Dengue virus is regularly introduced with infected humans returning from their travel to dengue endemic countries. However, there are no established populations of the principal vector, *Ae. aegypti* nor of *Aedes albopictus*, in the Netherlands, although *Ae. albopictus* has been regularly introduced, but intercepted (28). The current European geographic distributions of these mosquito species are shown in Figure 5. The climate is suitable for establishment of *Ae. albopictus* in Northwestern Europe, but is unsuitable for *Ae. aegypti*. Expansions of the latter in Europe is expected, but even with climate change it is not anticipated to establish in Northwestern Europe, where the temperature will remain unsuitable (29). Upon establishment of *Ae. albopictus*, the risk status will change from context 4 to 2. The current Dutch policy is therefore to prevent establishment of vectors by active surveillance and control using biocides at high-risk areas, since exotic mosquitoes are frequently imported.

Context 3 deals with VBDs that already have established putative vector populations, but so far there is no evidence of either pathogen circulation nor pathogen introductions. Surveillance is therefore focused on detecting the introduction or circulation of the disease causing pathogen as early as possible to enable adequate preventive and control measures. This may necessitate surveillance of the reservoir or sentinels (which may be humans or animals depending on the disease) and vector pyramids. In instances, where human cases of vector-borne zoonoses have been detected earlier than pathogen circulation in an enzootic cycle, human surveillance needs to be added to the surveillance strategy. In other cases, the availability or costs of samples determines the focus of surveillance. For all diseases belonging to this context, monitoring on the geographic distribution of the diseases in Europe is advisable.

**Table:** 

**West Nile fever risk in the Netherlands (Context 3)** Since human cases (humans as well as horses are dead-end hosts Figure 4B) can only occur following virus amplification cycles between bird-biting mosquitoes and birds. Given that endemic mosquitoes are putative WNV vectors, potentially infected populations of both birds (the host) and mosquitoes (the vector) are targeted for surveillance in high-risk areas suitable for transmission from an environmental/climatic point of view. Upon detection of an enzootic cycle of WNV, adequate preventive measures including public awareness campaigns, veterinary horse vaccination campaigns, and mosquito control activities may be implemented. In the Netherlands, a combined mosquito and WNV surveillance in the Oostvaardersplassen was started in 2009 and continued in 2010 with a wider screening for arboviruses, namely WNV, Usutu virus, Sindbis virus, Tahyna virus, and Batai virus (20, 30) and potential vector composition (30). In 2012, dozens of wild songbirds that were found dead in the Netherlands were tested for Usutu virus, but found negative (31). Up to now, no evidence of mosquito-borne virus circulation has been found in the Netherlands. Cerebrospinal liquors of humans with encephalitis of unknown cause were tested for the presence of flavivirus (32, 33). In collaboration with the Dutch Animal Health Service, a similar survey was performed on horses. In the UK, routine mosquito surveillance detected the presence of an additional WNV mosquito vector, *Culex modestus* in north Kent. To ensure an updated risk assessment on WNV for the UK, enhanced surveillance for the mosquito, and virus testing in mosquitoes and birds was implemented. The mosquito has shown evidence of expansion, but there is so far no evidence of the virus (34).
**Tick-borne encephalitis risk in the Netherlands (Context 3)** While its main vector, the sheep tick *I. ricinus*, occurs commonly, no autochthonous cases of tick-borne encephalitis (TBE) has been reported, nor any evidence of this virus circulating in an enzootic cycle in Netherlands. A closely related tick-borne flavivirus, named louping ill virus (LIV), however, is causing disease, predominantly in animals in upland areas of the UK and Ireland (35). In comparison with mosquito-borne disease outbreaks, the consequences of pathogen introduction in a region with a competent tick vector population are generally much more transient. In endemic areas, the permissive locations for transmission of tick-borne encephalitis virus are very local and patchy. In addition, the transmission of the virus between vectors occasionally occurs through co-feeding of immature stages of the tick on a non-viremic rodent host. Consequently, the detection of the pathogen soon after introduction is difficult and requires a very costly and intensive surveillance system. Recent publications suggest that animals that are neither a reservoir nor a sensitive host can act as sentinels for the circulation of tick-borne encephalitis virus, as shown with dogs and deer in Belgium (11, 12). However, the fact that a safe human vaccine against tick-borne encephalitis exists enables public health authorities to take effective preventive measures upon detection the first human case to protect the population for additional human cases, but not to prevent circulation. Such surveillance strategy aiming at an early detection of the first human case may be the most cost-effective one.

Context 2 includes VBDs with enzootic circulation of the pathogen, which so far has not resulted in reported human cases in the country and VBDs with frequent introduction of infectious reservoirs into the country, which so far has not resulted in autochthonous human cases. The presence of an established vector population for both categories is a requirement within this context.

**Table:** 

**Malaria risk in the Netherlands (Context 2)** The indigenous mosquito species, *Anopheles atroparvus*, is capable of transmitting malaria parasites *Plasmodium vivax*, but transmission has ceased in the second half of the last century through reduction of the vector population, changes in farm management, improved health, and eradicating the pathogen (36). In recent years, intense mosquito nuisance caused by *An. plumbeus*, a putative vector for *Plasmodium falciparum*, has been reported regularly in local agricultural area in the Netherlands and Belgium. Until recently, this species had been breeding only in tree holes, but has adapted to new breeding ground, namely manure gutters under abandoned pig stables in Netherlands and Belgium (37). Although human cases are imported, there is no autochthonous transmission to humans. The chance of malaria transmission is much more unlikely than arboviral transmission given the non-zoonotic nature of malaria. While dengue cases are infectious before or even without developing symptoms, patients infected with *falciparum*-malaria become infectious only after developing symptoms, which permits an opportunity to prevent subsequent transmission.
**Non-*Borrelia* tick-borne disease risk in the Netherlands (Context 2)** The sheep tick *I. ricinus* is responsible for the vast majority of human tick bites. In ticks in the Netherlands, a total of six (groups of) pathogens can be distinguished. For four groups, no association with human cases has been found (yet) (Table 2) (38). The latter may be due to one of the following causes. First, humans do not develop symptoms when infected with these pathogens (in this case, microorganisms would be the correct term). Second, humans do develop symptoms when infected with these pathogens, but these symptoms are mild and go unnoticed. Third, humans do develop symptoms when infected with these pathogens, but these symptoms are atypical and ambiguous and diagnosed under another cause. Fourth, humans do develop symptoms when infected with these pathogens, but these infections are travel-related. *Rickettsia helvetica*, can infect humans, but whether it causes symptoms and therefore disease burden is unclear.

**Table 2:** **Pathogens found in Dutch *I. ricinus* ticks**.

Pathogen	*I. ricinus* (n)	Tick infection rate (%)	Dutch human cases reported
*Borrelia burgdorferi* s.l.	628 (5308)	11.8	Yes
*Rickettsia helvetica*	1265 (4061)	31.1	No
*Anaplasma phagocytophilum*	44 (5343)	0.8	No
*Babesia spp*.	112 (4238)	2.0^b^	No
*Neoehrlichia mikurensis^a^*	300 (5343)	5.6	No
*Borrelia miyamotoi^a^*	6 (300)^b^	2.7	Yes

*^a^New to the Netherlands*.

*^b^tested in pools*.

Context 1 deals with VBDs that result in human cases or disease burden every year (Context 1a) or infrequently (Context 1b) (Table 1). In the Netherlands, as mentioned earlier, there are two tick- and no mosquito-borne diseases belonging to these contexts. To halt the increase in disease incidences, management (control and intervention) strategies for one or more of the three components (disease burden, pathogen, and vector) and the environment are important.

**Table:** 

***Borrelia miyamotoi* risk in the Netherlands (Context 1b)** In 2012 in the Netherlands, a tick-borne disease switched from context 2 to context 1b, as a result of a case of meningoencephalitis caused by relapsing fever spirochete *Borrelia miyamotoi* (9). Since there are no diagnostics available yet, the possibilities for surveillance are limited. Development of diagnostics has been prioritized to help to investigate the form of the human disease surveillance pyramid. Possibly the disease belongs to context 1a, but until evidence is provided it will be assigned to context 1b.
**Lyme disease risk in the Netherlands (Context 1a)** For the majority of Europe, including the Netherlands, Lyme disease belongs to context 1a. Because of the large and growing number of human infections, the epidemiology, ecology, and prevention of Lyme borreliosis is receiving vast public, political, and scientific interest in the Netherlands (14, 39–43). The strong increase in incidence has several biological, environmental, and societal reasons. The two main reasons for the increase are increases in the level of exposure of humans to ticks and increases in the abundance of infected ticks (44). Environmental change factors are also exacerbating the risk (45).

## Pan-European Context

Public health authorities are required to prepare for future threats and call for predictions of the likely changes in public health risks. Usually, they focus their preparedness on their own geographical region. Whereas, the context of a disease is essential to design a surveillance strategy, assessing the context of VBDs is actually an issue for many public health authorities as the necessary information is not always readily available. In other words, in some cases surveillance data are required to assess the context, in which the subsequent surveillance strategy is prioritized. In addition, for all diseases belonging to contexts 3–5, national health experts are required to monitor the geographic distribution of the diseases, pathogens, and vectors in Europe.

**Figure 6:**
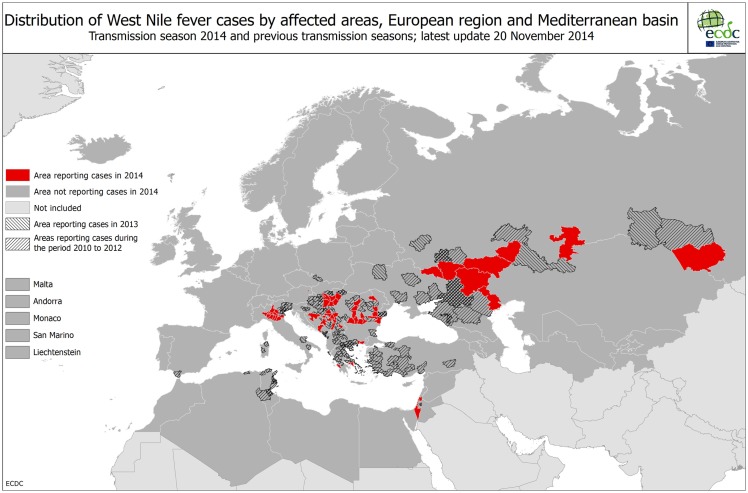
**West Nile fever map of Europe (Source: ECDC)**.

To assist EU member states in this task, the European Centre for Disease Prevention and Control (ECDC) runs various programs to aggregate data to develop Pan-European maps on vector distribution and VBD incidence, to identify drivers of change and to provide guidance (10, 45–48) (Figures 5 and 6). Based on such data, assessing the context of a particular VBD in each European country seems to be a rather straightforward task. Central databases, however, on infections of wildlife with vector-borne pathogens are lacking or incomplete, making the assessment of the pathogens category difficult. When ignoring this category, context 2 and 4 are effectively omitted from the Pan-European maps on VBD contexts, hindering appropriate risk assessments on national and international level. Through the Eden FP6 and Edenext FP7 programs, huge steps have been made with collecting data and identifying drivers for pathogen circulation (49). Following on from data collected on human cases and vectors, the aggregation of data on pathogen circulation in reservoir species is the final step in developing Pan-European maps of VBDs, based on their national context.

## Conclusion

Cataloging all possible surveillance activities on VBDs without putting these into context does not assist public health authorities with assessing comparative risk. Here, we propose a way forward for public health authorities to assess potential surveillance approaches for VBD based on its context and on a country-by-country basis. Surveillance efforts for VBDs that are endemic have a different focus and structure than those for VBDs that do not pose any immediate risk as neither the pathogen nor vector is present. Within the context of a VBD, the best surveillance strategy depends on the potential prospects for action and the costs/benefit analysis. This is particularly important given economic constraints, and therefore, a focus on interventions that achieve the largest health gain per euro spent seems eminently appropriate. For some VBDs, taking action prior to a VBD becoming an issue is preferable. Once a decision for intervention to decrease the disease burden (or group/category of diseases) or to mitigate a threat has been made, surveillance should be implemented in order to measure the effectiveness of this intervention (10).

It has become apparent that an interdisciplinary approach to the prevention and control of zoonoses is invaluable. Cross-sector working also ensures better preparedness and contingency planning, more efficient and effective surveillance systems, cost-sharing between sectors according to the benefits of control, increased health equity and improved sharing of logistics and costs for service provision (3). The field of integration of animal and human disease surveillance is new, but growing (4). Since the transmission cycles of several zoonotic pathogens occur in nature, involvement of stakeholders involved in environmental management and biodiversity-enhancing strategies is the next logical step. This requires an extension of the data collection approaches to further enhance disease intelligence (2) rather than a preset static structure. The described contextual surveillance for VBD extended with veterinary and wildlife health along with public health is highly applicable in a One Health approach.

## Conflict of Interest Statement

The authors declare that the research was conducted in the absence of any commercial or financial relationships that could be construed as a potential conflict of interest.
